# Rückblick auf das erste „Corona-Semester“. Ergebnisse einer semesterbegleitenden Untersuchung der Task Force Digitale Lehre des Instituts für Politische Wissenschaft und Soziologie der Universität Bonn

**DOI:** 10.1007/s41358-020-00243-2

**Published:** 2020-11-25

**Authors:** Manuel Becker, Felix Leßke, Enrico Liedtke, Eva Hausteiner, Christiane Heidbrink, Jakob Horneber, Tim Huyeng, Shushanik Minasyan, Hendrik W. Ohnesorge, Maximilian Raths, Penelope Wessel

**Affiliations:** Institut für Politische Wissenschaft und Soziologie, Rheinische Friedrich-Wilhelms-Universität Bonn, Lennéstr. 25, 53113 Bonn, Deutschland

## Einführung: Die Herausforderung sozialwissenschaftlicher Lehre im Online-Semester

Als im Kontext der COVID-19-Pandemie das öffentliche Leben massiv eingeschränkt wurde, sah sich auch das Hochschulwesen mit ganz neuen Anforderungen konfrontiert. Im März 2020 zeichnete sich ab, dass das Sommersemester unter keinen Umständen im „Normalbetrieb“ ablaufen würde, wobei die Reaktionen zunächst zögerlich waren: Inmitten einer dynamischen und präzedenzlosen Lage konnten die Universitätsleitungen den Lehrenden nur langsam und schrittweise mitteilen, unter welchen Bedingungen – und ab wann – im Sommersemester unterrichtet werden sollte. Ressourcen sowie Potenziale des eLearning mussten recht zügig abgewogen und Werkzeuge für innovative krisenresistente Lehrszenarien gefunden werden.

Schnelles Handeln in der Umstellung von Präsenz- zu Online-Lehre war also gefragt, um „eine Gratwanderung zwischen schneller Reaktion und Qualität zu meistern“ (Tovar 2020). Der Technologieeinsatz stellte im hochschuldidaktischen Bildungsprozess plötzlich ein unverzichtbares Element dar, und vorhandene Präsenzlehrkonzepte mussten über Nacht digitalisiert werden. Über Jahre eingespielte Prozesse griffen plötzlich nicht mehr: Fest geplante Seminare, Vorlesungen und Übungen wurden angepasst und „umgemodelt“, und obwohl die Universitätsleitungen auf Hochtouren an technischen Infrastrukturen für eine kontaktlose Lehre arbeiteten, existierte lange kein klarer Fahrplan für den Corona-Sommer. Dementsprechend wurden dezentral sehr unterschiedliche Lehrmodelle – synchron, asynchron oder *blended*, Video‑, Podcast- oder textbasiert, – in Erwägung gezogen, unterschiedliche Softwares und Plattformen verglichen und getestet, Datenschutzbedenken geprüft und Home-Office-Arbeitsplätze aufgerüstet. Diese kurzfristige mediendidaktische Transformation, die eine qualitativ ausreichende Vermittlung von Lerninhalten ermöglichen sollte, war insbesondere für jene Lehrenden herausfordernd, die in ihren bisherigen Veranstaltungen auf technische Unterstützung sowie Kommunikationswege verzichtet hatten. Hinzu kam die Herausforderung, digitale Prüfungsformen zu entwerfen oder Präsenzprüfungen anzupassen: Raumkapazitäten, Prüfungsgerechtigkeit sowie Datenschutzkonformität mussten jeweils berücksichtigt werden (Hochstrat und Oemisch 2020).

Im Vergleich zu Universitäten in den USA oder im europäischen Ausland verfügten die deutschen Institute zwar über den „Luxus“, die Lehre nicht im laufenden Betrieb, sondern in den Semesterferien digital umstellen zu müssen. Das machte die Situation allerdings nur geringfügig besser, denn bereits der etablierte, prä-pandemische Lehralltag war von Defiziten geprägt, die sich unter den neuen Bedingungen weiter auswirkten: zu große Kurse, die Abdeckung eines beträchtlichen Teils der Lehre durch befristet Beschäftigte und externe Lehrbeauftragte – und vor allem, gerade im Vergleich zu Universitäten in den USA, Großbritannien oder den Niederlanden, ein relativ ausgeprägter Digitalisierungsrückstand. Nicht zu vernachlässigen ist die Tatsache, dass die Krisensituation die Lehrenden ins Homeoffice zwang, was die Vereinbarkeit des Berufsalltags und der Familie grundsätzlich vor neue Herausforderungen stellte. Entwicklung eigener Prozesse und viel Selbstorganisation waren angefragt, um mit den vielfältigen Anforderungen sowie den andauernden Wechseln erfolgreich umgehen zu können (Neubert 2020). Eher wenige Lehrende verfügten, Stand April 2020, über eine umfassende Vertrautheit mit der jeweiligen universitären Online-Lernplattform oder mit ergänzenden Tools, und die Serverkapazitäten der Rechenzentren erwiesen sich recht schnell als begrenzt: Bis dato war universitäre Lehre auch am Institut für Politische Wissenschaft und Soziologie der Universität Bonn immer als Präsenzlehre mit meist geringen digitalen Elementen durchgeführt worden.

Die zentrale Herausforderung lautete also: Wie lässt sich eine digitale Lehre auf die Beine stellen, die zwar nicht zur Dauerlösung werden soll, aber temporär den Studienfortschritt der Student*innen ohne große qualitative Einbußen sicherstellen kann – und dabei auch inklusiv darauf achtet, dass trotz der physischen Distanz keine Student*innen außen vor bleiben? Wie kann diese Umstellung insbesondere in kurzer Frist und unter Bedingungen knapper Ressourcen und begrenzter zeitlicher Möglichkeiten gelingen?

Zu Beginn des „Digitalsemesters“ gingen die Einschätzungen darüber sehr weit auseinander. Auf der einen Seite forderte ein offener Brief, erstunterzeichnet von knapp 1400 Hochschullehrer*innen ein „Nichtsemester“, womit nicht der Ausfall der Lehre gemeint war, sondern eine Mäßigung der Ansprüche an und Fristverlängerungen für Student*innen und Mitarbeiter*innen unter den erschwerten Pandemiebedingungen (Offener Brief 2020a). Auf der anderen Seite warnten Professor*innen vor einem Dammbruch durch die Gewöhnung an digitale Lehre und einem dauerhaften Verlust der Universität als „Ort der Begegnung“ (Offener Brief 2020b; Scholz 2020).

In den Feuilletons wurden diese und andere Einschätzungen breit debattiert, wobei zunächst ein tendenziell skeptischer Tonfall dominierte. Immer wieder warnten Kommentare wie auch Interviews mit Hochschulbeschäftigten vor einem Verlust an Qualität, einer dauerhaften Kürzung der ohnehin schon knappen Ressourcen wie auch vor einer Beschädigung einer offenen und gemeinsamen Lehr- und Lernatmosphäre (Stubner 2020). Nur vereinzelt waren Stimmen zu hören, die die erzwungene Präsenzpause auch als Chance zu werten versuchten – etwa, um sich weiter mit den Möglichkeiten digitaler Tools auch im Rahmen der künftigen Präsenzlehre vertraut zu machen,^1^ aber auch, um zeitweise die Vorteile einer reinen Online-Lehre zu nutzen.^2^ Die Devise lautete hier, die kaum vermeidliche Situation zu akzeptieren und das Beste aus ihr zu machen. Aus der Perspektive der Politikwissenschaften und der Soziologie stellte sich dabei in erster Linie die Frage, wie in der beschleunigten Digitalisierung des Wissenschaftsalltags produktive Seminardiskussionen in offener, inklusiver Atmosphäre gelingen könnten (Fleuß 2020).

In Vorbereitung eines solchen prüfenden Blicks haben sich die Lehrenden am Bonner Institut für Politische Wissenschaft und Soziologie – insbesondere aus dem Mittelbau – zu Beginn des Sommersemesters 2020 früh in einer Task Force zur Digitalen Lehre koordiniert, deren Ziel nicht allein der Austausch über *best practices* zur Verbesserung der gerade stattfindenden Kurse war, sondern auch eine aufmerksame Beobachtung der gemachten Erfahrungen, um Schlüsse für die künftige Lehre zu ziehen. Die in der Task Force behandelten Themen reichen von der notwendigen technischen Unterstützung bis hin zu didaktischen Fragen, wie etwa Inhalte bestmöglich vermittelt werden und das Stattfinden einer kritischen Diskussion auch und gerade auf Basis digitaler Lehrformate garantiert werden kann. Gefragt ist hier vor allem die systematische Auseinandersetzung mit vielen Akteur*innen, indem die Impulse von den Lehrenden, Student*innen sowie universitären Einrichtungen wie Rechenzentren zusammengetragen und ausgewertet werden.

Da das erste reine Digitalsemester nun hinter uns liegt, erscheint die Gelegenheit günstig, eine diesbezügliche Zwischenbewertung zu wagen.^3^ Nicht nur pandemiebedingt wird das Thema Online-Lehre die deutschen Universitätsinstitute weiter beschäftigen. Auch „nach Corona“ wird zu prüfen sein, welche Praktiken und Tools sich bewährt haben und wie die Erfahrungen von 2020 sich auf verschiedene Aspekte und die Erfahrungen von Lehrenden und Student*innen ausgewirkt haben.

Hierzu sollen, ausgehend von den Erfahrungen des Sommersemesters, im Folgenden zunächst die verschiedenen digitalen Tools für Lehr- und Lernformen vorgestellt sowie in ihren Vor- und Nachteilen bewertet werden. Ein besonderer Schwerpunkt wird hierbei auf dem Videokonferenzanbieter Zoom liegen (2). Anschließend werden die Ergebnisse einer Online-Umfrage unter den Lehrenden des Instituts präsentiert (3) und an einer entsprechenden Befragung der Student*innen (4) gespiegelt. Abschließend werden die wesentlichen Erfahrungen aus dem vergangenen Sommersemester mit Blick auf die kommenden Monate in einem kurzen Fazit gebündelt (5).

## Vorstellung digitaler Tools für Lehr- und Lernformen

Sozialwissenschaftliche Lehre lebt besonders von der Diskussion – also unmittelbarer Aktion und Reaktion zwischen den Teilnehmer*innen. In der digitalen Lehre bietet sich dazu das Format der *Videokonferenz* an, durch das es am ehesten gelingt, eine typische Seminarsitzung zu simulieren. Im Corona-bedingt stark wachsenden Markt entsprechender Plattformen konnte sich das einfach zu bedienende US-amerikanische Portal *Zoom* in kürzester Zeit als Referenz etablieren.^4^

Neben dem virtuellen Konferenzraum ist es mit weiteren Kommunikationsfunktionen ausgestattet, die sich gerade im Lehrkontext als notwendig erweisen. Denn noch mehr als Sitzungen mit physischer Präsenz bedürfen Videokonferenzen verstärkt interaktiver Momente, um die Aufmerksamkeit auf einem hinreichenden Niveau zu halten. Mit den verschiedenen Tools können Sitzungen abwechslungsreich gestaltet beziehungsweise gezielt mit sinnvollen Elementen abseits klassischer Redebeiträge ergänzt werden.

Über die Funktion des *Bildschirmteilens* lassen sich Referate, präsentationsgestützte Kurzvorträge o. ä. fast wie in Präsenz durchführen. Den Teilnehmer*innen wird das Anschauungsmaterial direkt auf ihrem Bildschirm angezeigt. Für die Sitzungsleitung empfiehlt sich hier die Nutzung zweier Monitore, um neben der Präsentation auch die Teilnehmer*innen im Blick zu haben. Dieses Tool eignet sich auch, um einzelne Dokumente, Grafiken, Videos oder andere Dateien zu zeigen und auch audiovisuell zugänglich zu machen. Eine weitere damit verknüpfte Funktion ist das *Whiteboard*, mit dem sich Tafelbilder erstellen lassen, die auch als Bilddatei abgespeichert werden können.

Sogenannte *Breakout Sessions* ermöglichen die Einrichtung von Arbeitsgruppen oder separierten Diskussionsräumen während der Konferenzsitzung. Sowohl die Gruppengröße als auch die Dauer der Unterbrechungen lassen sich individuell einstellen. Die Zuteilung der Teilnehmer*innen erfolgt manuell oder per Zufall. Per Chat kann die Sitzungsleitung Arbeitsaufträge in die einzelnen Gruppen schicken oder sich selbst hinzuschalten. Die Erfahrung zeigt, dass Teilnehmende in Kleingruppen eher bereit sind, die Videoübertragung zu aktivieren, während in größeren Runden viele Kameras ausgeschaltet bleiben.

Teilnehmende auch ohne Redebeiträge einzubinden kann über die *Chatfunktion* gelingen. Sie eignet sich für ein Brainstorming, das Sammeln von Antworten, aber auch, um komplexere Fragen oder Arbeitsaufträge sichtbar zu machen. Der Chat bietet eine gute Ergänzung, Fragen seitens der Teilnehmenden auch während längerer Redebeiträge zu ermöglichen oder zu lange Redelisten zu vermeiden. Gleichzeitig kann er beispielsweise genutzt werden, um Links für eine sitzungsbegleitende Schnelllektüre oder als weiterführende Hinweise zu verschicken.

Zur weiteren Auflockerung der Videokonferenz können *Umfragen* genutzt werden. Sie eignen sich, um Einschätzungen oder Meinungen zu bestimmten Sachverhalten einzuholen, Fragen zur Vorbereitungslektüre zu stellen oder den Wissenstand der Student*innen zu testen. Die Umfragen müssen im Vorfeld der Sitzung erstellt und können während der Sitzung in beliebiger Reihenfolge aufgerufen und auch wiederholt werden. Die Ergebnisse der Abstimmungen lassen sich für alle Teilnehmenden sichtbar machen. Wird ein Konferenzraum auf Dauer angelegt, bleiben auch die einzelnen Umfragen erhalten und können wiederholt genutzt werden (z. B. zur Evaluierung des Lernfortschritts). Einfache Stimmungsbilder oder Abstimmungen lassen sich durch Reaktionstools wie Ja‑/Nein-Abstimmungen über den Chatkanal oder Emoticons durchführen.

Neben Videokonferenzen gibt es eine Fülle von Funktionen digitaler Lernplattformen, die nach gewünschtem Lehr- und Lernziel zum Einsatz kommen können, und hier in Auswahl vorgestellt werden sollen:

Ein *Forum* bietet die Möglichkeit, Diskussionsthemen anzulegen. Hierbei sehen Student*innen die Beiträge aller Kursteilnehmender und können direkt auf diese antworten. Daher sollten Gesprächsregeln im Vorfeld besprochen und festgelegt werden, um eine fachlich angemessene Diskussion zu gewährleisten. Aufgrund des dynamischen Gesprächscharakters verringert sich die Übersichtlichkeit, sodass es in jedem Fall einer Moderation und einer Zusammenfassung am Ende bedarf. Das *Forum* zeichnet sich durch seinen niedrigschwelligen Zugang aus. Es erfordert nur wenige Voreinstellungen, bietet jedoch viele Optionen wie etwa die Einbindung von Bildmaterial. Es lässt sich zudem als Fragenforum für fachliche, formale oder inhaltliche Schwierigkeiten nutzen – mit dem Vorteil, dass sowohl Lehrende als auch Mitstudent*innen antworten können. Je nach Lernplattform und Voreinstellung können Beiträge sogar anonym eingestellt werden. Auf diese Weise sinkt die Hemmschwelle, Fragen zu stellen.

Für eine übersichtliche Pro-Kontra-Diskussion eignet eine etwa von der Webseite *Kialo.com *angebotene Diskussionsplattform. Zwar müssen sich alle Teilnehmende zunächst auf der Webseite registrieren und sich mit der komplexen Funktionsweise vertraut machen, hierzu bietet die Seite aber einen Support und Tutorials an. Die Bildungssparte von *Kialo *erlaubt, Diskussionen nur eingeladenen Nutzerinnen und Nutzern sichtbar zu machen. Das Tool zeichnet sich dadurch aus, dass die Diskutierenden Beiträge anderer kommentieren und bewerten können. Sehr gut bewertete Argumente werden als erstes angezeigt. Die Diskussionspfade werden zudem visuell nachgezeichnet, sodass es jederzeit möglich ist, zum übergeordneten Diskussionspunkt zurückzukehren.

Eine gemeinsame Diskussion lässt sich auch über ein *Wiki* führen. Diese Form des kollaborativen Schreibens ermöglicht eine übersichtliche Ergebnissicherung, wobei der Diskussionsgrad geringer ist. In einem Wiki entsteht üblicherweise kein Gespräch, sondern eine Informationssammlung, in der alle Beitragenden Änderungen am Text vornehmen können. Die Zuordnung der Beiträge zu einzelnen Personen ist daher nur eingeschränkt möglich. Technisch sind Wikis anspruchsvoll – sowohl in Einrichtung als Nutzung. Sie bieten allerdings den Vorteil, dass am Ende des Nutzungsprozesses ein ausformuliertes und übersichtlich strukturiertes Ergebnis steht, das über eine Downloadfunktion auch als PDF exportierbar ist. Wikis sind prinzipiell für die simultane Bearbeitung mehrerer Personen geeignet. Sollten die Kapazitäten der universitären Plattformen nicht für simultanes Schreiben ausreichen, lassen sich schriftliche Ergebnisse mit kooperativen Texteditoren wie *Edupad* fixieren und anschließend einfügen. Diese Tools bieten außerdem die Vorteile, dass die Beiträge unterschiedlicher Autor*innen durch farbliche Markierungen erkennbar bleiben, sie technisch sehr einfach zu bedienen sind und keine Einzelregistrierung erfordern.

Wenn allerdings statt einer Diskussion zu einer Fragestellung die individuelle oder gruppenbezogene Leistungsstandüberprüfung im Vordergrund steht, bieten sich Formate an, die das Einreichen und Auswerten von Übungsaufgaben möglich machen. *ILIAS-gestützte E‑Learning-Plattformen* stellen einen entsprechenden Funktionsumfang etwa unter der Bezeichnung *„Übung“* zu Verfügung. Dort ist es möglich, Aufgabenstellungen mit Abgabefrist und automatischen Erinnerungen zu definieren. Mittels einer Peer-Feedback-Funktion lässt sich außerdem innerhalb des Kurses ein offenes oder anonymes Feedback eintragen. Anhand nach der Abgabe verfügbarer Musterlösungen können Student*innen im Anschluss an die Abgabe direkt ihren Leistungsstand überprüfen. Lehrende wiederum haben die Möglichkeit, eine Liste aller Abgaben abrufen und somit den Wissensstand vergleichen zu können. Zwar ist die technische Konzeption seitens der Lehrenden komplexer als in den vorherigen Fällen, dagegen ist eine *Übung* auf Seiten der Student*innen leicht zu bedienen.

Für alle genannten Funktionen stehen verschiedene Hochschullernplattformen zur Verfügung, deren technischer Umfang allerdings variiert (Tab. 1).

**Table 1:** 

Funktion	Vorteil	Nachteil
Zoom	Einfache Bedienbarkeit und leichter Zugang Erleichtert direkte Kommunikation (Diskussion, Fragen etc.) Schafft nahezu Seminaratmosphäre Interaktive Ergänzungstools (Formal) höhere Teilnehmerzahl als in Präsenzsitzungen	Stabile Internetverbindung mit ausreichend Datenvolumen notwendig Hardwarebedingte Qualitätsverluste (z. B. Mikrophon/Tonqualität, Bildschirmgröße/Unübersichtlichkeit) Leichteres „Ausklinken“ der Teilnehmenden möglich Soziale Interaktion geht vor und nach der Sitzung verloren
Forum	Niedrigschwelliger Einstieg Hoher Diskussionsfaktor Hohe Interaktivität Moderatorenfunktion Bewertungsfunktion für Themen	Risiko der Unübersichtlichkeit Zentrale Lernergebnisse müssen ggfs. zusammengefasst werden Diskussionsregeln müssen festgelegt und überprüft werden
Wiki	Hohe Übersichtlichkeit Exportfunktion Verlaufsspeicherung Kommentarfunktion Bewertungsfunktion für einzelne Seiten	Variierender Diskussions- und Interaktivitätsgrad Einstiegsschwierigkeit Umfangreiche Funktionen Anonymität
Übung	Einfache Bedienbarkeit für Student*innen Kriterienkataloge und Musterlösungen definierbar (Peer‑)Feedback- und Bewertungsfunktion Fristsetzung möglich Liste aller Abgaben einsehbar	Niedriger Diskussionsfaktor Geringe Interaktivität Kein allgemeines Lernergebnis Komplexe Voreinstellungen

## Auswertung einer Umfrage unter den Lehrenden

Um einen umfassenderen Eindruck der Erfahrungen mit den verschiedenen digitalen Tools zu gewinnen, wurde zum Ende des abgelaufenen Sommersemesters eine Befragung unter den Dozent*innen des Instituts für Politische Wissenschaft und Soziologie der Universität Bonn durchgeführt. Die Befragung wurde als Online-Stichprobe über das Befragungstool Questback konzipiert. Der Link zur Teilnahme wurde am 15. Juli 2020 verschickt, am 3. August 2020 erfolgte die Versendung eines Reminders. Eine Beantwortung war bis zum 31. August 2020 möglich. Der Fragebogen enthielt sowohl geschlossene, als auch offene Frageblöcke, die ein breites Abbild der Erfahrungen der Dozent*innen aus dem Sommersemester 2020 zeichnen sollten. Insgesamt wurde die Befragung von 46 von 78 Lehrpersonen ausgefüllt.

Zunächst wurde in dem Fragebogen die Frage nach dem Lehrformat/den Lehrformaten gestellt, welche(s) von den Befragten im vergangenen Semester in digitaler Form abgehalten worden ist/sind. Dabei gaben 36 Befragte an, ein oder mehrere Seminare und sieben weitere ein oder mehrere Blockseminar(e) gegeben zu haben. „Vorlesung“ wurde an dieser Stelle nur einmal genannt. Auch Lehrformate wie Kolloquien, Forschungspraktika und Übungen wurden maximal zwei Mal genannt. Aufgrund dieser deutlich höheren Fallzahlen an Seminaren sollen im Folgenden vorrangig die Ergebnisse für dieses Lehrformat zusammengefasst werden.

Ferner wurde nach den verwendeten Unterrichtsmedien gefragt. Bis auf eine Person gaben alle Befragten an (*n* = 45), Zoom verwendet zu haben. Darüber hinaus verwendeten 34 Befragte die universitätseigene E‑Learning-Plattform *eCampus*. Acht Personen erstellten für ihre Lehre Videocasts, vier weitere Podcasts. Zudem wurde in der Freitextkategorie dreimal die Webcloud *Sciebo* genannt, sowie jeweils einmal *Loom* und *Scrumblr*.

Die Qualität der digitalen Lehre in den Seminaren (*n* = 36) im Vergleich zur Präsenzlehre wurde von knapp zwei Dritteln der befragten Dozent*innen als gleichwertig beurteilt (18 Personen bei 29 gültigen Antworten), neun Personen gaben an, dass die digitale Lehre schlechter gewesen sei, zwei Personen stuften sie sogar besser ein. Hinsichtlich der Art, wie die Seminare abgehalten wurden, zeigt sich ein recht diverses Bild, wobei der Anteil an synchroner, durch Videokonferenzen organisierter, Lehre überwiegt. So gaben sieben Personen an, dass sie ausschließlich, und 13 Personen, dass sie überwiegend synchrone Lehre abgehalten hätten. Eine gleichwertige Mischform beider Formate wurde von acht Personen gewählt. Auch wenn zwei Personen überwiegend asynchrone Lehre abgehalten haben, wurden in allen Fällen synchrone Elemente in der Lehre genutzt. Dies unterstreicht die bereits berichtete sehr starke Nutzung von Zoom in der Lehre unter allen Befragten.

Für die Seminare hatten sich im Durchschnitt etwa 25 Personen angemeldet, wobei die Spanne von fünf Personen bis 40 Personen reichte. Die Anwesenheit der Student*innen in den Seminaren wurde als tendenziell überdurchschnittlich wahrgenommen: Etwas weniger als die Hälfte der Befragten berichtete von einer durchschnittlichen Anwesenheit in den Seminaren (13 Personen von 30 gültigen Antworten), ein Drittel sah die Anwesenheit als eher hoch an (10 Personen) und fünf Befragte stuften die Anwesenheit als sehr hoch ein. Nur zwei Personen gaben an, eine geringe Anwesenheit erlebt zu haben. Ähnlich positiv gestalten sich die Aussagen über die Motivation der Student*innen. Hier gab etwas mehr als die Hälfte eine durchschnittliche Motivation an (16 Personen von 30 gültigen Antworten), vier Personen eine eher hohe und sechs Personen eine sehr hohe Motivation. Von Seiten der Student*innen wurde vornehmlich ein positives Feedback zurückgemeldet. Zwölf Dozent*innen gaben an, dass die Rückmeldungen der Student*innen hinsichtlich ihrer Lehrmethoden sehr positiv gewesen seien, bei 15 Befragten war sie eher positiv. Nur zwei Personen berichteten ein neutrales Feedback.

Besonders Video-Kommunikationsplattformen wie Zoom kommt in der digitalen Lehre eine wichtige Bedeutung zu, da Sie das entscheidende Werkzeug für synchrone Lehrformate darstellen. Auch hier wurden die Erfahrungen mit der Lehre über Zoom in der Studie abgefragt und fielen durchweg positiv aus: 16 Personen gaben an, den Einsatz von Zoom in der Lehre als sehr gut zu bewerten, 22 Personen empfanden diesen als eher gut (von insgesamt 38 gültigen Antworten).

Die als positiv wahrgenommene Umsetzung der digitalen Lehre fordert allerdings durchaus an anderer Stelle Ihren Tribut, war die Umstellung für die Lehrenden doch mit großem Aufwand verbunden. Im Vergleich zu vorherigen Semestern gaben 13 Befragte den zeitlichen Aufwand für die Vorbereitung der Lehre als sehr viel höher an, 20 als eher höher. Nur zwei Personen hatten einen vergleichbaren zeitlichen Aufwand. Eine Zeitersparnis durch die digitale Lehre ergab sich in der Vorbereitung für keinen der Befragten. Dies spiegelt sich auch in der Forschungsarbeit wider. In Anlehnung an eine Befragung des Wissenschaftsverlags De Gruyter fragten wir danach, welche Auswirkung die digitale Lehre auf die Forschungsarbeit gehabt hat. Dort hatte etwa die Hälfte der Befragten angegeben, durch die Auswirkungen des Coronavirus weniger zum Schreiben gekommen zu sein als zuvor (De Gruyter Conversations 2020). Als Grund dafür gaben 68 % die Online-Lehre an. In unserer Befragung fiel das Ergebnis sogar noch deutlicher aus. Nur drei Befragte gaben an, im Sommersemester 2020 durch die digitale Lehre eher mehr Zeit für die Forschung gehabt zu haben, vier Personen hatten etwa die gleiche Zeit zur Verfügung. Hingegen hatten 16 Personen „eher weniger“ und neun „sehr viel weniger“ Zeit für Forschungsarbeit. Zudem gaben zwei Personen an, dass andere Gründe sie vom Forschen abgehalten hätten.

Die Auswertung der Antworten auf die offenen Fragen kann sich ebenfalls nur auf das Lehrformat der Seminare beschränken, da das Sample für Übungen und Vorlesung für eine qualitative Auswertung zu klein ist. Grundsätzlich sind die Ergebnisse vor allem geprägt durch Berichte von überrascht positiven Erfahrungen mit der plötzlich neuen Situation der digitalen Lehre. So waren nur wenige Dozent*innen bereits vorher mit den neu einzusetzenden Tools vertraut und dennoch wurde durch erhebliches Eigenengagement und zeitlichen Aufwand die Lehre häufig innerhalb von weniger als 2 Monaten mit großem Erfolg auf den digitalen Lehrbetrieb umgestellt. Negativ vermerkten die Dozent*innen vor allem eine im Vergleich zur Präsenzlehre deutlich verschlechterte Diskussionskultur in den Zoomsitzungen. So wurde der direkte Austausch und die Ansprechbarkeit nicht zuletzt durch ausgeschaltete Kameras als stark eingeschränkt wahrgenommen. Dies lässt auch die überdurchschnittlichen Anwesenheitswerte in einem differenzierteren Licht erscheinen: Im Vergleich zum Normalbetrieb quantitativ hohen Teilnehmer*innenzahlen steht eine qualitativ vergleichbare oder sogar geringere aktive Beteiligung gegenüber. Die Passivität der Student*innen wurde dabei durch Scheu, mangelnde menschliche Beziehungen zwischen den Studierenden wie auch zu den Dozent*innen und technische Hindernisse (Internetverbindung, keine Kamera vorhanden etc.) begründet. Die fehlende direkte Resonanz der Student*innen wurde ebenfalls moniert. So fiel es schwer einzuschätzen, „ob das angestrebte Niveau adäquat für die Student*innen ist, welche Themen mehr Aufmerksamkeit verdient hätten und welche zusätzlichen Interessen von den Student*innen noch angesprochen werden sollten“ (Fbn. 56). Die fehlende Interaktion mit den Student*innen, beschrieben als „lifeless as images“ (Fbn. 92) und „das Gefühl eine Wand zu unterrichten“ (Fbn. 47) war deshalb vor allem in den Zoom-gestützten Seminaren für viele Dozent*innen eine Herausforderung. Die „berühmten schwarzen Kacheln“ (Fbn. 43, 54, 56, 72) waren dabei im Verhältnis zur Gruppengröße tendenziell vor allem bei größeren Seminaren ein Problem.

Auch die hohen Anforderungen an die Aufmerksamkeit der Dozent*innen durch die Gleichzeitigkeit von mehreren Kommunikationskanälen (Wortbeiträge, Handzeichen, Chatbeiträge, laufende Präsentationen) wurden „generell als anstrengender und aufzehrender“ (Fbn. 56) als die Präsenzlehre eingestuft. Dies spiegelt noch einmal die bereits im quantitativen Teil berichtete Mehrbelastung der Dozent*innen wider. Weiterhin wurden Zweifel am Datenschutz von Zoom geäußert (vgl. Fbn. 47, 50, 63), die auch nicht durch die breit kommunizierte Nutzung der Server des universitätsinternen Rechenzentrums ausgeräumt werden konnten. Trotz dieser Schwierigkeiten wurde der Einsatz synchroner Elemente der Lehre im Vergleich zu asynchronen als erfolgreicher eingestuft. Dies zeigte sich auch bereits in den oben beschriebenen statistischen Daten zur Häufigkeit der Nutzung synchroner Lehre. Es gab aber auch Stimmen, die sich für eine Kombination aus beiden Formaten aussprachen und damit die zeitliche Flexibilität von asynchronen Anteilen mit der pädagogisch wertvollen direkten Interaktion von synchroner Lehre zu vereinen versuchten.

In großer Breite wurde aber auch von positiven Erfahrungen mit der neuen, digitalen Lehre berichtet. So wich die anfängliche Skepsis schnell der Experimentierfreude und einer in den Antworten auch immer wieder wahrzunehmenden Begeisterung über die freigesetzten Potentiale der neuen Lehrformate. Der angestrebten Kompensation der negativen Effekte räumlicher Entfernung näherte man sich dabei über ständige Anpassungen und Verbesserung auch noch während des Semesters weiter an. So konnte das Problem erhöhter Scham für Wortbeiträge im Plenum durch Break-Out-Sessions innerhalb von Zoom vermindert und durch kleinere wöchentliche Studienleistungen der Austausch zwischen Studierenden und Dozent*innen im digitalen Studium erhöht werden.

Aufseiten der Studierenden wurde eine große Bereitschaft, sich auf die neuen Formate einzulassen, beobachtet, wenn etwa von den Dozierenden eine „überdurchschnittlich gute Vorbereitung der Studierenden“ (Fbn. 34) konstatiert wurde. Gerade durch die Möglichkeit Studienleistungen zu diversifizieren, „wurde sehr viel kreatives Potential freigesetzt“ (Fbn. 40). So wurden verschiedenste Varianten von Studienleistungen in der Lehre eingesetzt, etwa: Podcasts, Essays, Exzerpte, Poster, Online-Glossar, wöchentliche Beantwortung von Übungsaufgaben, Teilnahme am Forum, Positionspapiere, Rezensionen, Diskussionsreferate, Sitzungsprotokolle. In der synchronen Lehre mit Zoom wurde zudem mehrfach das implementierte Tool der Umfragefunktion gelobt, da hiermit eine „hohe Aktivierung der Studierenden“ (Fbn. 68) erreicht werden konnte. Dabei wurde angemerkt, dass „Meinungs- und Erfahrungsabfragen“ gewinnbringender seien als Wissensfragen im Stil von „Wer-wird-Millionär“ (vgl. Fbn. 56).

Weiterhin wurde die im Digitalen deutlich unkompliziertere Zuschaltung von Fachexperten und Kollegen von anderen Standorten hervorgehoben. So konnte auf spezifische Interessen der Student*innen über die Zuschaltung eines*r Gastreferent*in teilweise sogar kurzfristig noch eingegangen werden. Die technischen Schwierigkeiten durch die Zoom-Umgebung wären dabei sogar geringer als „beim Anschluss der Technik im Seminarraum“ (Fbn. 76). Die amerikanische Videokonferenzsoftware wurde dabei generell als „technisch zuverlässig“ (Fbn. 83) und „intuitiv“ (Fbn. 40) beschrieben. Gerade auch durch die diversen Möglichkeiten an den Sitzungen teilzunehmen (dies ist bspw. auch telefonisch möglich) wurde „weitestgehend Vollinklusion“ (Fbn. 56) ermöglicht. Eine Person lobte auch die Whiteboard-Funktion, die eine Archivierung der Gruppenergebnisse ermöglichte. Die fast kanonisch gewordene Form der Power-Point-Präsentation wurde allerdings als für die digitale Lehre (noch) unvorteilhafter eingeschätzt, da vor allem stark reproduktive und auf reine Wissenspräsentation angelegte Präsentation nicht zur nötigen „Aufmerksamkeit“ führten und im digitalen Unterricht die Gefahr der Ablenkung deutlich höher sei. Dagegen wurden große Erfolge mit Video- und Podcasts erzielt, die die Student*innen bereits vor den Sitzungen hochluden und rezipierten, um dann in der Seminarstunde selbst mehr Zeit für die direkte Interaktion zu haben. Allerdings sahen einige Dozent*innen die Methodenpluralität in den verschiedenen Seminaren auch kritisch, da sich die Student*innen immer wieder auf ein neues Tool einlassen müssten und leicht den Überblick verlieren könnten. Solche Schwierigkeiten müssten sich ggf. in der im nächsten Kapitel vorgestellten Student*innenbefragung widerspiegeln. Ähnlich wurden die Tendenzen zu einer „Verschulung“ des Studiums durch „Deadlines“ (Fbn. 32) und einen „Zwang zur Partizipation“ (Fbn. 51) im „digitalen Semester“, mit Blick auf die Student*innen und die Institution Universität Bonn, in ihrer Ambivalenz zwischen Leistungssteigerung und Bevormundung auch kritisch betrachtet.

Aufgefordert ein Gesamtfazit zu ziehen, betonten die meisten Dozent*innen eher die positiven Momente der erfolgreichen Bewältigung der Herausforderung. Mit gesteigerter „Experimentierfreudigkeit und viel Motivation auf allen Seiten“ (Fbn. 34) ließe sich doch auf ein „gelungenes Semester“ zurückblicken. Zoom sei dabei zu „mehr als nur einer Notlösung“ (Fbn. 51) geworden und der allgemeine Tenor entsprach der Freude über die neu erlernten digitalen Kompetenzen, die in Zukunft auch in der Präsenzlehre eingesetzt werden könnten. Zwar darf man mit Blick auf die Aussagen der Dozent*innen am Institut für Politische Wissenschaft und Soziologie in Bonn festhalten, dass, bei allem Erfolg, die digitale Lehre den Präsenzbetrieb nicht ersetzen kann und man – sobald es das Infektionsgeschehen zulässt – die Rückkehr in die Seminarräume begrüßen würde, solange dies allerdings nicht möglich ist, lässt sich wohl eine der Antworten auf die letzte Frage unserer Umfrage als Devise ausgeben: „Weitermachen, weniger jammern“ (Fbn. 65).

## Auswertung einer Umfrage der Fachschaft

Ein stimmiges Gesamtbild über die Erfahrungen mit der digitalen Lehre erfordert neben der Dozent*innen- auch die Student*innensicht. Hierfür können zwei im Verlauf des Sommersemesters 2020 über den Instagram-Kanal der Fachschaft Politik & Soziologie der Rheinischen Friedrich-Wilhelms-Universität durchgeführte Umfragen unter den Social-Media-Nutzer*innen herangezogen werden. Verwendet wurde das Format der sogenannten „Storys“, deren Besonderheit es ist, dass sie für nur 24 h aufrufbar sind. Somit war die Möglichkeit der Teilnahme an den beiden Umfragen jeweils auf 24 h beschränkt. Die Ergebnisse der Umfragen sind nicht repräsentativ für alle Student*innen, da nur Nutzer*innen der App „Instagram“ teilnehmen konnten. Allerdings erreicht der Instagram-Kanal der Fachschaft mit stetig ungefähr 500 Followern einen Großteil der ca. 1600 Fachstudent*innen, sodass die Umfragen einen wesentlichen Einblick in die studentische Wahrnehmung der digitalen Lehre vermitteln können.

Die erste Umfrage wurde am 5. Mai 2020 und somit zu Beginn des Semesters durchgeführt und bestand aus sechs Fragen, wobei drei offen und drei geschlossen formuliert waren: Zunächst wurden die Student*innen gefragt, ob sie den Online-Lehrbetrieb am Institut für Politische Wissenschaft und Soziologie als gut umgesetzt empfanden. Von den 159 Nutzer*innen, die auf die Frage reagiert haben, stimmten 120 für „Ja“ und 39 für „Nein“. Kritisiert wurden vor allem die Art und Weise der zu erbringenden semesterbegleitenden Studienleistungen sowie der gestiegene Arbeitsaufwand, zum Teil aber auch grundsätzlicher die digitale Durchführung des Semesters als solche. Besonders wurde bemängelt, dass die Studienleistungen zu wenig an die digitale Lehre angepasst seien. Die Nutzung des Programms „Zoom“ zur Ausführung der digitalen Lehre wurde von den Student*innen positiv aufgenommen, da dadurch ein persönlicher Kontakt zwischen Student*innen und Dozent*innen ermöglicht wurde. Es wurde jedoch angemerkt, dass eine Variation zwischen synchroner und asynchroner Lehre von Vorteil wäre, um allen Student*innen die gleichen Chancen zur Teilnahme zu ermöglichen. Weiterhin abgefragt wurde der Umfang der Studienleistungen. Von 162 Nutzer*innen empfanden 60 den Umfang als angemessen und 102 als zu viel. Die Nutzung der E‑Learning-Plattform der Universität „eCampus“ wurde von einer großen Mehrheit (136 Personen) als sinnvoll erachtet. Lediglich 18 Personen hielten diese nicht für nicht sinnvoll.

Weiterhin erfragte die Fachschaft, welche konkreten Themen an die Lehrenden des Instituts herangetragen werden sollten. Dabei wurden erneut die zu umfangreichen Studienleistungen sowie der zu hohe Arbeitsaufwand genannt. Außerdem wurde eine bessere Übersicht der Foren auf „eCampus“ gewünscht. Schließlich wurden positive Rückmeldungen zur Umsetzung der digitalen Lehre erbeten. Dabei wurde vor allem die gute und schnelle Umstellung von Präsenz- auf Digitale Lehre sowie die Kommunikation von Seiten des Instituts – gerade im Vergleich zu anderen Instituten – ausdrücklich gelobt. Ebenfalls hervorgehoben wurden die teils neuen Formate der Studienleitungen sowie die Aufstockung der vorhandenen Online-Literatur. Positiv wurde außerdem das Engagement und die Hilfsbereitschaft vieler Dozent*innen wahrgenommen.

Die zweite Umfrage fand nach Ende der Vorlesungszeit am 9. August 2020 statt. Diese bestand aus zehn Fragen, von denen fünf geschlossen und fünf offen formuliert waren:

In der ersten Frage wurde erneut danach gefragt, ob die Student*innen den Online-Lehrbetrieb im Sommersemester 2020 als gut umgesetzt empfunden haben. Darauf antworteten ebenfalls eine deutliche Mehrheit (98 Personen) mit „Ja“, lediglich 21 Nutzer*innen verneinten eine gute Umsetzung. Nach einer Begründung für die negativen Eindrücke gefragt, bemängelten die Befragten vor allem die fehlende Abwechslung, die fehlende Innovationsbereitschaft einzelner Dozent*innen sowie einen zu hohen Leseaufwand. Letztgenannter Kritikpunkt wird auch durch die Antworten auf den erfragten Vergleich des Arbeitsaufwands im digitalen Sommersemester mit vergangenen Präsenzsemestern bestärkt: So bezeichneten 119 Befragte den Aufwand als im Vergleich höher und lediglich 17 Personen als geringer. Neben dem – möglicherweise einmaligen – Aufwand, sich mit den technischen Gegebenheiten neuer Plattformen und Funktionen vertraut zu machen, schlägt hier vermutlich auch die noch fehlende Erfahrung, mit wie viel Arbeit die Nutzung der neuerprobten Medien und Aufgabenstellungen tatsächlich verbunden ist, zu Buche.

Insgesamt werden allerdings viele Möglichkeiten der digitalen Lehre als hilfreich angesehen. Als besonders wertvolle Elemente einer digitalen Lehre wurden vorrangig die wöchentlichen Zoom-Sitzungen sowie die neu genutzten Lehr- und Lernformate wie beispielsweise Podcasts und Videos genannt. Generell wurden Zoom, aber auch die universitätseigene E‑Learning-Plattform als besonders hilfreiche Software zur Unterstützung der digitalen Lehre angegeben.

Bei der Frage welche Unterrichtsform die Student*innen bevorzugten (synchron oder asynchron), ergibt sich ein geteiltes Bild: 79 der Befragten votierten für synchron, 50 für asynchron. Für beide Varianten wurden dabei schlagkräftige Argumente vorgebracht: Als Argument für synchrone Lehre wurde unter anderem genannt, dass diese am ehesten einer normalen Seminaratmosphäre entspräche und somit unter anderem mögliche Fragen besser geklärt werden können. Die asynchrone Lehre zeichne sich hingegen besonders durch eine Förderung des selbstständigen und eigenverantwortlichen Lernens aus. Entsprechend wurde allgemein eine Kombination beider Formen als beste Lösung angesehen. Diese Erkenntnisse unterstreichen die auch in der Lehrendenbefragung geäußerte Präferenz für gemischte Formate.

Dazu passend wurde dafür plädiert, auch in die Lehre der künftigen Semester digitale Elemente zu integrieren. Besonders die Nutzung von Lehrvideos und Podcasts sowie die angebotene größere Diversität der Studienleistungen wurden als positiv und erhaltenswert beurteilt. Beibehalten werden soll – nach Ansicht einiger Befragter – nach Möglichkeit auch die schnellere Kommunikation mit den Dozent*innen.

Insgesamt lässt sich festhalten, dass die meisten Student*innen zu Beginn des Semesters noch viel Verständnis für Unklarheiten, Übergangslösungen und verspätete Informationen aufbrachten. Gerade mit Blick auf die kurze Vorbereitungszeit wurde die Umstellung auf die digitale Lehre und die dadurch stattfindende Erweiterung möglicher Lehrmethoden und semesterbegleitend zu erbringende Leistungen am Institut durchaus positiv beurteilt. Gleichzeitig zeigen die erwähnten Kritikpunkte allerdings auch, dass es noch Verbesserungspotential dabei gibt, die neuen Lehrmöglichkeiten sinnvoll, passfähig und verbunden mit einem realistisch zu bewältigenden Arbeitsaufwand einzusetzen.

## Abschluss und Ausblick

Was bleibt vom „digitalen Sommersemester 2020“ und welche Lehren sind für die Zukunft zu ziehen? Nach einer Phase der pandemiebedingten Ungewissheiten, mit denen sich wie alle gesellschaftlichen Lebensbereiche auch die akademische Welt konfrontiert sah, stellte das Bonner Institut für Politische Wissenschaft und Soziologie mit Beginn des Sommersemesters 2020 zügig die Weichen auf digitale Lehre. Startschwierigkeiten, bedingt durch kurzfristig geänderte Anforderungen an die Lehrplanung und zumindest teilweise fehlende Vertrautheit mit den entsprechenden Möglichkeiten wichen im weiteren Verlauf des Semesters der zunehmend routinierten Verwendung digitaler Lehrformen. Programme und Werkzeuge wie Zoom, Podcasts, oder Wikis hielten Einzug in den Lehralltag und sind heute kaum noch wegzudenken.

Der regelmäßige kollegiale Austausch im Rahmen der eingerichteten Task Force Digitale Lehre hat früh dazu beigetragen, Schwierigkeiten auszuräumen und Best-Practice-Beispiele zu identifizieren. Eine unter allen Dozent*innen am Institut durchgeführte Umfrage nach Ende der Vorlesungszeit ergab nicht zuletzt aus diesem Grund ein weitgehend positives Bild: Mit klarem Fokus auf synchron durchgeführte Lehre, insbesondere durch Verwendung der Videokonferenz-Software Zoom, wurde das Semester weitestgehend als Erfolg gewertet. Die beiden von der Fachschaft durchgeführten Umfragen zu Beginn und nach Ende der Vorlesungszeit kamen zu ähnlichen Ergebnissen. Bereits bei der ersten Umfrage im Mai, also kurz nach Beginn der Lehrveranstaltungen, bewerteten sogar mehr als 75 % der Befragten den Umstieg auf digitale Formate als gut gelungen.

Im Verlauf des Semesters, als der erste Einstieg ins Digitale geglückt war und der Umgang mit digitalen Formaten vertrauter wurde, zeigten sich vielfach gar Experimentierfreude und Kreativität bei der Entdeckung und Nutzung der unbestrittenen Potentiale digitaler Lehre. Dass der recht abrupte Umstieg auf digitale Formate erfolgreich gestaltet werden konnte, ist dabei keinesfalls nur den Mitarbeiter*innen aus Hochschulrechenzentrum und Verwaltung sowie den Dozent*innen am Institut zu verdanken, sondern insbesondere auch allen Student*innen, die, das zeigen die Einschätzungen studentischer Anwesenheit, Motivation und Mitarbeit in den Lehrveranstaltungen, die neuen Umstände ebenso aufgeschlossen annahmen und die digitale Lehre erfolgreich mitgestalteten.

Die unter den Dozent*innen durchgeführte Umfrage zeigt jedoch auch negative Seiten der digitalen Lehre auf: weniger Zeit für eigene Forschungsarbeit sowie, traditionell ein elementarer Bestandteil sozialwissenschaftlicher Lehre, eine leidende Diskussionskultur sind hier besonders zu nennen. Vergleichbar damit attestierten auch die Umfragen unter den Student*innen einen erheblichen Mehraufwand im „digitalen Semester“. Deutlich wurde zudem der Wunsch nach mehr Diversifizierung der verwendeten Lehrformen, sowohl bei der Gestaltung der wöchentlichen Sitzungen als auch bei der Erbringung von Studienleistungen.

Diese Befunde mögen einerseits den auf beiden Seiten fehlenden oder zumindest kargen Erfahrungen im Umgang mit den entsprechenden Tools geschuldet sein – und zukünftige (digitale) Semester versprechen aus eben diesem Grunde Besserung. Allerdings werden wohl auch in Zukunft bestimmte Komponenten bei einer Verlagerung der Lehre ins Digitale auf der Strecke bleiben. Spontanität, das Gespür für das richte Lehrtempo und die unkomplizierte Nachfrage im Seminarraum lassen sich trotz aller Möglichkeiten nicht in Gänze digitalisieren. Die Teilnahme an digitaler Lehre ist (1) zudem abhängig von einwandfreien technischen Voraussetzungen (u. a. moderne Hardware, stabile Internetverbindung), die den ohnehin noch nicht hinreichend gleichwertigen Zugang zur Hochschule und die Partizipationschancen darin weiter einschränken; und behindert (2) den Abbau habitueller Hürden durch das Fehlen sozialer Interaktion zwischen den Veranstaltungen und das Eindringen in den privaten Raum (Videokonferenz) während der Lehrzeiten.

Weitere Praxis, und die sie begleitende Forschung und Evaluation, werden nötig sein, um die Potenziale digitaler Lehre noch besser kennen- und nutzen zu lernen. Die Task Force Digitale Lehre wird daher auch weiterhin aktiv bleiben und das kommende Wintersemester wird neue Chancen und ganz eigene Herausforderungen bieten: Einerseits bestehen längere Vorbereitungszeiten und mittlerweile auch gewachsene Vertrautheit mit digitalen Lehrformen. Andererseits stellt das Wintersemester auch neue Aufgaben; ist es doch traditionell das Semester, an dem Studienanfänger*innen ihr Studium aufnehmen und entsprechende Einführungsveranstaltungen angeboten werden. Gerade aus diesem Grund wird es wichtig sein, aufbauend auf den nun gemachten Erfahrungen, die digitale Lehre fortlaufend zu optimieren und um weitere Inhalte und Angebote zu erweitern.

Abschließend sei auf einen Aspekt hingewiesen, der für die Zukunft der Universität im digitalen Zeitalter von besonderer Bedeutung ist: Die Corona-Pandemie hat eine Rosskur eingeleitet und gewissermaßen über Nacht zu einer Zwangsdigitalisierung der deutschen Universitätslandschaft geführt. Lange Zeit aufgeschobene und teilweise auch verschlafene Entwicklungen wurden unter dem Druck äußerer Entwicklungen beherzt angegangen und konnten auch weitestgehend erfolgreich gestaltet werden. In dieser Hinsicht hat die Pandemie die Wirkung eines Katalysators entwickelt, dessen positive Auswirkungen auf den Digitalisierungsgrad deutscher Hochschulen nicht von der Hand zu weisen sind. Hier gilt es, Bewährtes zu erhalten und in die Zeit „nach Corona“ zu überführen.

Eine Universität jedoch ist weit mehr als die Summe der an ihr durchgeführten Forschungsarbeiten und angebotenen Lehrveranstaltungen, ob nun im digitalen oder im traditionellen Präsenzlehrbetrieb. Sie war seit ihrer Erfindung und Verbreitung als Institution im europäischen Mittelalter auch stets ein Marktplatz revolutionärer Ideen, ein Forum freien Austausches und ein Ort kontroverser Diskussion – und dies ausdrücklich nicht nur im Seminarraum. Zwischen den Lehrveranstaltungen blieb Zeit, das Gehörte und Gelernte im Kreise der Kommiliton*innen zu vertiefen; in Freistunden im Café oder – im Bonner Falle – im Hofgarten zu hinterfragen; und selbst das gemeinsame Mittagessen in der Mensa diente zu weit mehr als der bloßen Nahrungsaufnahme. Vielmehr ist das studentische und kollegiale Miteinander aller Angehörigen einer Hochschule seit jeher eine tragende Säule universitären Lebens – und in einer Zeit der zunehmenden politischen und gesellschaftlichen Polarisierung, zu der auch Phänomene wie Echo-Kammern und Filter-Blasen ihren Beitrag leisten, ist diese Funktion heute vielleicht wichtiger als je zuvor. Eine Digitalisierung der Lehre kann daher nur dann nachhaltig von Erfolg gekrönt sein, wenn sie nicht auf Kosten der genannten Komponenten erfolgt. Sie ist folglich eher als ein ergänzendes, denn als ein (vollständig) ersetzendes Element erfolgreicher Hochschullehre zu betrachten.

## Footnotes

1 Vgl. die bereits sechs Jahre zurückliegende Bedarfsdiagnose bei Höppner et al. (2014).

2 Zur Debatte siehe Busen und Huhnholz (2020).

3 Erste Einschätzungen: Zierer (2020a, 2020b), sowie Sommer (2020).

4 Neben Zoom, das sich einiger Kritik u. a. zum Datenschutz stellen musste, gibt es auch alternative Angebote wie z. B. Big Blue Button, Jitsi Meet oder Webex. Aus Sicht der Nutzerfreundlichkeit bietet es sich jedoch an, instituts- oder gar universitätsweit eine einheitliche Software zu nutzen.
